# Age-Related Hearing Loss Is Accelerated by Repeated Short-Duration Loud Sound Stimulation

**DOI:** 10.3389/fnins.2019.00077

**Published:** 2019-02-27

**Authors:** Juan Carlos Alvarado, Verónica Fuentes-Santamaría, María Cruz Gabaldón-Ull, José M. Juiz

**Affiliations:** Instituto de Investigación en Discapacidades Neurológicas (IDINE), Albacete, Spain Facultad de Medicina, Universidad de Castilla-La Mancha, Albacete, Spain

**Keywords:** presbycusis, sensorineural hearing loss, noise-induced hearing loss, evoked potentials, auditory brainstem responses

## Abstract

Both age-related hearing loss (ARHL) and noise-induced hearing loss (NIHL) may share pathophysiological mechanisms in that they are associated with excess free radical formation and cochlear blood flow reduction, leading to cochlear damage. Therefore, it is possible that short, but repeated exposures to relatively loud noise during extended time periods, like in leisure (i.e., musical devices and concerts) or occupational noise exposures, may add to cochlear aging mechanisms, having an impact on the onset and/or progression of ARHL. Consequently, the aim of the present study was to determine if repeated short-duration overexposure to a long-term noise could accelerate permanent auditory threshold shifts associated with auditory aging in an animal model of ARHL. Toward this goal, young adult, 3-month-old Wistar rats were divided into two groups: one exposed (E) and the other non-exposed (NE) to noise overstimulation. The stimulation protocol consisted of 1 h continuous white noise at 110 dB sound pressure level (SPL), 5 days a week, allowing 2 days for threshold recovery before initiating another stimulation round, until the animals reached an age of 18 months. Auditory brainstem response (ABR) recordings at 0.5, 1, 2, 4, 8, 16, and 32 kHz were performed at 3, 6, 12, and 18 months of age. The results demonstrate that in the E group there were significant increases in auditory thresholds at all tested frequencies starting already at 6 months of age, which extended at 12 and 18 months. However, in NE animals threshold shifts were not evident until 12 months, extending to 18 months of age. Threshold shifts observed in the E animals at 6 and 12 months were significantly larger than those observed in the NE group at the same ages. Threshold shifts at 6 and 12 months in E animals resembled those at 12 and 18 months in NE animals, respectively. This suggests that repeated noise overstimulation in short-duration episodes accelerates the time-course of hearing loss in this animal model of ARHL.

## Introduction

Hearing loss is the most frequent sensory impairment and, globally, the fourth largest source of disability in the population of all ages (Mathers et al., 2000, 2008; World Health Organization, 2018. It affects approximately 466 million people (6.1% of the world’s population), and unless appropriate measures are taken, its incidence could rise up to 630 million by 2030 and to 900 million by 2050 (World Health Organization, 2017b, 2018). Hearing loss has a profound impact on the individuals and their social environments leading to a decrease in quality of life (World Health Organization, 2017b, 2018). It also impacts economy, affecting several sectors including occupational, educational and health care, with an estimated annual global cost over US$ 750 billion (World Health Organization, 2017b, 2018). All this highlights the need to continue studying the causes of hearing loss, in order to establish strategies for optimal therapeutic approaches. Among the different possible causes, noise and aging are the most common etiological factors in the development of hearing loss in the adult population (World Health Organization, 2006, 2017b). Although, individually noise and aging are important enough to be considered a global issue, they usually coexist and interact increasing the worldwide burden.

Noise-induced hearing loss (NIHL) is the consequence of overexposure to loud noise, characterized by a permanent increase in auditory thresholds (permanent threshold shift, PTS) (Clark and Bohne, 1999; World Health Organization, 2006; Daniel, 2007; Le Prell et al., 2007b; Basner et al., 2014). There is a consensus in that noise is defined as any unpleasant sound (Berglund et al., 1999;  Śliwińska-Kowalska and Davis, 2012), and its source coming mainly from occupational activities related to transportation and industries (World Health Organization, 1997, 2006; Daniel, 2007; European Commission, 2008;  Śliwińska-Kowalska and Davis, 2012; Basner et al., 2014). However, it is important to note that some “pleasant” sounds, under certain circumstances, also may lead to NIHL. Thus, sources of loud sound that might induce hearing loss could originate from leisure activities such as the use of personal portable musical devices, playing with loud toys, attending sports or musical events, or fitness classes (World Health Organization, 1997, 2006; Daniel, 2007; European Commission, 2008;  Śliwińska-Kowalska and Davis, 2012; Basner et al., 2014).

Age-related hearing loss (ARHL) or presbycusis in the human clinic, is a gradual and irreversible age-dependent decline of the auditory function, which is reflected in a progressive increase in auditory thresholds, mainly in the high frequency range. Subjects present degraded frequency discrimination and limitations in speech comprehension tasks (Boettcher, 2002; Syka, 2002; Gordon-Salant, 2005; Bielefeld et al., 2010; Huang and Tang, 2010; Sprinzl and Riechelmann, 2010; Fetoni et al., 2011). ARHL is one of the most frequent sensory disabilities in the elderly, affecting more than 33% of the population over 60 years old (Mathers et al., 2000, 2008; Gopinath et al., 2009; Lin et al., 2011; Yamasoba et al., 2013). Since it has been estimated that this aged population will increase in global rates from 12 to 22% between 2015 and 2050 (World Health Organization, 2017a), the number of affected people in the next three decades will increase substantially. The reason that there is no effective medication against ARHL, is due to the fact that its etiopathogenesis is multifactorial and very complex and still remains unclear (Huang and Tang, 2010; Fetoni et al., 2011; Yamasoba et al., 2013; Melgar-Rojas et al., 2015b; Alvarado et al., 2018).

It has been suggested that “common pathogenic pathways” are shared by NIHL and ARHL (Alvarado et al., 2015, 2018; Tavanai and Mohammadkhani, 2017). For instance, excess free radical build-up in the cochlea, due to increased metabolic demands after noise overstimulation, metabolism dysregulation associated with age as well as reduction in cochlear blood flow, seem to play key roles in the etiopathogenesis of both NIHL and ARHL (Mills and Schmiedt, 2004; Henderson et al., 2006; Le Prell et al., 2007a,b; Bielefeld et al., 2010; Huang and Tang, 2010; Schmiedt, 2010; Fetoni et al., 2011; Shi, 2011; Lee, 2013; Yamasoba et al., 2013; Fujimoto and Yamasoba, 2014; Alvarado et al., 2015; Melgar-Rojas et al., 2015b). As aforementioned, the synergistic interaction between NIHL and ARHL involves a complex “pathogenic continuum” of coincident, overlapping or independent mechanisms converging in sensorineural hearing loss (Bielefeld et al., 2010; Fetoni et al., 2011; Walling and Dickson, 2012; Melgar-Rojas et al., 2015b; Parham et al., 2015). Nevertheless, despite the fact that there is a great deal of information about noise and aging, there is still no consensus on the long-term functional effects of noise exposure on ARHL (Fetoni et al., 2011). Moreover, since most of these studies are based on a single noise exposure or short-term noise stimulation protocols and humans are exposed daily and for a long time to a variety of loud sounds, additional research is needed to better mimic these conditions. Therefore, the aim of the present study was to evaluate the possible impact that overexposure to relatively loud sounds of short duration repeated over an extended time period may have on the onset and/or progression of presbycusis in an animal model of ARHL.

## Materials and Methods

### Animals

Sixteen adults male Wistar rats (Charles River, Barcelona, Spain), were housed at the Universidad de Castilla–La Mancha animal facility (Albacete, Spain), under controlled conditions of temperature (22–23°C) and humidity (60 ± 5%), and on a 12 h light/dark cycle and food/water *ad libitum*. All procedures were approved by the Ethics Committee on Animal Experimentation at the University of Castilla-La Mancha (Permit Number: PR-2013-02-03) and were conformed to Spanish (R.D. 53/2013; Law 32/2007) and European Union (Directive 2010/63/EU) regulations for the care and use of animals in research.

### Noise Overstimulation Protocol

**FIGURE 1:**
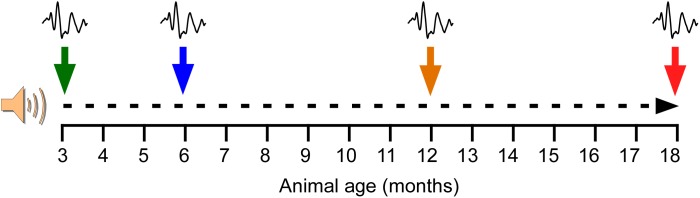
Noise overstimulation protocol. Following the baseline ABR recordings prior to any noise exposure at the age of 3 months, the noise exposure sessions began. The noise overstimulation protocol consisted of a continuous white noise (110 dB SPL) for 1 h a day for 5 days, with 2 days of recovery before initiating the next stimulation round, until the animals reached 18 months. Additional ABR recordings were performed at 6, 12, and 18 months of age. At these time-points, the recordings in the exposed animals were performed after the 2 days of recovery and right before initiating the corresponding round of 5 days of noise overstimulation.

In order to evaluate the effects of repetitive, short-duration noise overstimulation on aging, animals were distributed into two main groups: (1) a non-exposed group (NE, *n* = 8) and (2) an exposed group (E, *n* = 8) to noise overstimulation. The noise overstimulation protocol consisted of a continuous white noise at an intensity of 110 dB sound pressure level (SPL), starting at 3 months of age (Figure 1). Following the baseline ABR recordings prior to any noise exposure at the age of 3 months, the noise exposure sessions began at 1 h per day for 5 consecutive days, followed by 2 days of “recovery” before initiating another stimulation round. This protocol was repeated until the animals reached the age of 18 months. The sound was delivered inside a methacrylate reverberating chamber with 60 × 70 × 40 (length × width × height) cm with non-parallel and tilted walls to avoid standing waves and ensure a more homogeneous sound field. The chamber was located into a double wall sound–attenuating booth placed inside a sound–attenuating room.

ABR recordings in both NE and E animals were performed first at 3 months of age (NE3 and E3, control condition), before beginning the noise overstimulation protocol in E rats, and then at 6 (NE6 and E6), 12 (NE12 and E12), and 18 (NE18 and E18) months of age (Figure 1). At these time points, the recordings in the exposed animals were performed after the 2 days of recovery and right before initiating the corresponding round of 5 days of noise overstimulation.

### Auditory Brainstem Response (ABR) Recordings

ABR recordings were performed as described elsewhere (Alvarado et al., 2012, 2014, 2016, 2018; Fuentes-Santamaría et al., 2012, 2013, 2014; Melgar-Rojas et al., 2015a). The rats were anesthetized with isoflurane (1 L/min O2 flow rate) at 4% and 1.5–2% for induction and maintenance; respectively. Then, the animals were placed into a sound-attenuating and electrically shielded booth (EYMASA/INCOTRON S.L., Barcelona, Spain) that was located inside a sound-attenuating room. During recordings, the temperature was monitored with a rectal probe and maintained at 37.5 ± 1°C using a non–electrical heating pad. After anesthesia, subdermal needle electrodes (Rochester Electro–Medical, Tampa, FL, United States) were placed at the vertex (non–inverting) and in the right (inverting) and left (ground) mastoids. Auditory stimulation and signal recordings were performed using a BioSig System III (Tucker-Davis Technologies, Alachua, FL, United States). Specifically, the sounds were generated digitally by the SigGenRP software (Tucker-Davis Technologies, Alachua, FL, United States) and the RX6 Piranha Multifunction Processor hardware (Tucker-Davis Technologies, Alachua, FL, United States), and were delivered into the external auditory meatus of the right ear using an EDC1 electrostatic speaker driver (Tucker–Davis Technologies) through an EC-1 electrostatic speaker (Tucker-Davis Technologies). The stimuli, consisted of pure tone bursts sounds (5 ms rise/fall time without a plateau with a cos2 envelope delivered at 20/s) at seven different frequencies (0.5, 1, 2, 4, 8, 16, and 32 kHz). Prior to recording, calibration was performed using SigCal software (Tucker-Davis Technologies) and an ER-10B+ low noise microphone system (Etymotic Research Inc., Elk, Groove, IL, United States). Auditory evoked potentials were filtered (0.3–3.0 kHz), averaged (500 waveforms) and stored for offline analysis.

### ABR Data Analysis

#### Auditory Thresholds

To determine auditory thresholds in NE and E animals, evoked responses were measured in 5 dB steps descending from 80 dB SPL. Background activity, measured before stimulus onset, was recorded. Next, for each of the frequencies tested, the auditory threshold was defined as the stimulus intensity that evoked waves with a peak–to–peak voltage greater than two standard deviations (SD) of the background activity (Cediel et al., 2006; Garcia-Pino et al., 2009; Alvarado et al., 2012, 2014, 2016, 2018; Fuentes-Santamaría et al., 2012, 2013, 2014; Melgar-Rojas et al., 2015a). The maximum level of intensity was established at 80 dB, in order to avoid possible acoustic trauma in NE rats and any additional noise overstimulation in E animals during the recordings (Gourévitch et al., 2009; Alvarado et al., 2012, 2014, 2016, 2018; Fuentes-Santamaría et al., 2012, 2013, 2014; Melgar-Rojas et al., 2015a). Following the noise overstimulation protocol in the E group, if no auditory evoked responses were obtained at 80 dB, the auditory thresholds were set at that value for statistical analysis (Subramaniam et al., 1992; Trowe et al., 2008; Alvarado et al., 2012, 2014, 2016, 2018; Fuentes-Santamaría et al., 2012, 2013, 2014; Melgar-Rojas et al., 2015a).

#### Threshold Shift

The threshold shift was calculated for each of the frequencies by subtracting the auditory thresholds at the different time-points evaluated (6, 12, and 18 months) from the auditory thresholds in the control condition (3 months) (Alvarado et al., 2012, 2014, 2016, 2018; Fuentes-Santamaría et al., 2012, 2013, 2014; Melgar-Rojas et al., 2015a).

#### Wave Amplitudes

Since waves I, II and IV are the largest and the most consistent waves in ABRs from Wistar rats (Church et al., 2010, 2012b; Alvarado et al., 2012, 2014, 2016, 2018; Fuentes-Santamaría et al., 2012, 2013, 2014; Melgar-Rojas et al., 2015a), all parameter measurements related to the evoked responses were performed on these three waveforms. The wave amplitude was defined as the sum of the absolute values of the positive peak and the subsequent negative trough of the waves (Popelar et al., 2008; Church et al., 2010, 2012b; Alvarado et al., 2012, 2014, 2016, 2018; Fuentes-Santamaría et al., 2012, 2013, 2014; Melgar-Rojas et al., 2015a).

#### Wave Amplitude Ratio

In order to normalize the measurement of the waves, the amplitudes were analyzed as absolute amplitudes and also as relative amplitudes using the wave amplitude ratio (Boettcher et al., 1996). The wave amplitude ratio was calculated as follows:

Ratio = (WATP/WACC)

Where WATP is the amplitude of the wave at each time-point and WACC is the wave amplitude in the control condition before the noise exposure.

#### Wave Latencies

As reported previously (Chiappa et al., 1979; Chen and Chen, 1991; Gourévitch et al., 2009; Alvarado et al., 2012, 2014, 2016, 2018), two wave latencies were evaluated: (1) the absolute positive latency (time in ms between the stimulus onset and the positive peak), and (2) the absolute negative latency (time between the stimulus onset and the negative trough). Furthermore, the positive and negative interpeak intervals between the waves I-II, II-IV, and I-IV were calculated. An acoustic transit time of 0.5 ms between the speaker’s diaphragm and the rat’s tympanic membrane was added to the positive and negative latencies.

#### Statistical Analysis and Preparation of Figures

All data are expressed as mean ± SEM. The measurements of the waves amplitudes and latencies were performed at 80 dB SPL unless otherwise indicated. Comparisons among the different groups were performed using two-way repeated measures analysis of variance (ANOVA) with noise overstimulation (non-exposed vs. exposed), as an independent variable and age (3, 6, 12, and 18 months), as a repeated independent variable. All the ABR parameters measured in the present study were the dependent variables. For each of the frequencies, the possible statistically significant main effect of the noise overstimulation and age was also evaluated. If the main analysis indicated a significant effect of one factor or an interaction between factors, a Scheffé *post hoc* analysis was made. Significance levels (α) and power (β) were set to 0.05 and 95%, respectively. Canvas (Deneba v6.0) software package was used for the preparation of figures.

## Results

### Auditory Thresholds

**FIGURE 2:**
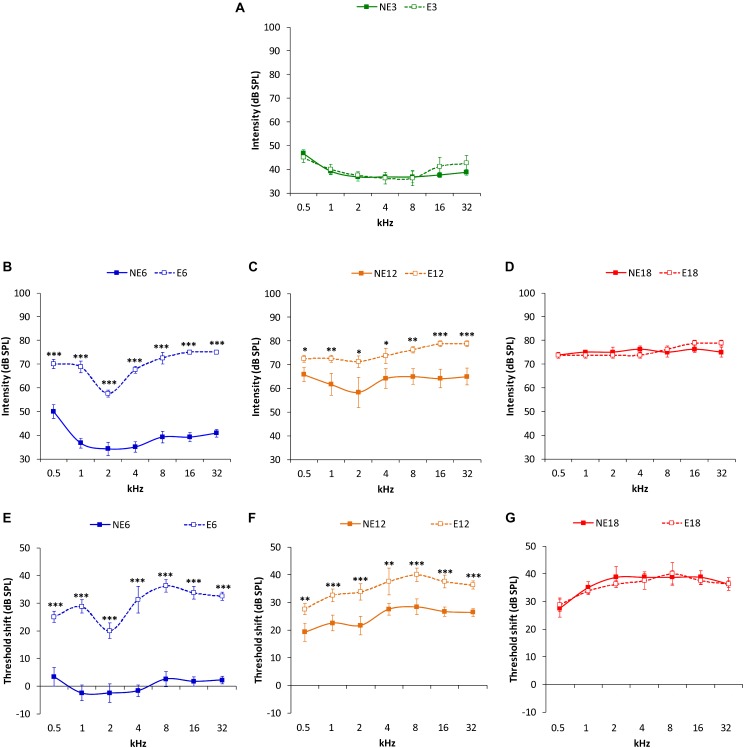
Line graphs illustrating auditory thresholds and threshold shifts at the different frequencies evaluated in NE and E rats at 3, 6, 12, and 18 months. In both NE3 and E3 animals, previous any noise exposure, the mean values of the auditory thresholds were similar **(A)**. At 6 months, the auditory thresholds **(B)** and the threshold shifts **(E)** in E6 rats were significantly higher than those found in the NE6 rats. Although, auditory thresholds **(C)** and threshold shifts **(F)** were elevated in both NE12 and E12 groups, these values were still statistically significantly higher in the E12 rats when compared to NE12 animals. At 18 months of age, no significant differences were detected between noise exposed and non-exposed rats **(D,G)**. ^∗^*p* < 0.05, ^∗∗^*p* < 0.01, ^∗∗∗^*p* < 0.001.

ABR recordings in 3 month-old rats in both the NE and E groups (Figure 2A) showed normal auditory thresholds, similar to those reported previously in the Wistar rat (Jamesdaniel et al., 2008; Church et al., 2010, 2012b; Alvarado et al., 2012, 2014, 2016, 2018; Fuentes-Santamaría et al., 2012, 2013, 2014; Pilati et al., 2012; Melgar-Rojas et al., 2015a). In the NE rats, no differences were observed in average threshold values between 3 (NE3) and 6 months (NE6). However, both in NE12 and NE18, there was a significant increase in auditory thresholds at all frequencies analyzed as a function of age (Figure 2A–D), which was consistent with previous findings (Alvarado et al., 2014, 2018). In contrast, in the E rats, an increase in auditory thresholds was observed already at 6 months of age (E6) (Figure 2B) and it persisted elevated in the E12 and E18 groups (Figure 2C,D). ANOVA revealed a significant effect of noise overstimulation and age on auditory thresholds [*F*(_3,__258)_ = 64.49, *p* < 0.0001]. Subsequent application of Scheffé´s *post hoc* test demonstrated that auditory thresholds in the E6 (Figure 2B) and E12 (Figure 2C) groups were statistically significantly higher at all assessed frequencies, when compared to those in age-matched NE rats and were similar to those observed in NE12 and NE18 rats, respectively (compare Figure 2B–D). In 18-month-old rats, no statistical differences were detected (Figure 2D).

ANOVA also showed a significant effect of both noise overstimulation and age on auditory thresholds shift [*F*(_2,__190)_ = 6.27, *p* < 0.001]. Scheffé´s *post hoc* test indicated that values in the E6 group were significantly higher when compared to the NE6 group (Figure 2E). Despite that threshold shifts in the NE12 rats were elevated, values were significantly lower than those found in E12 rats (Figure 2F). Finally, there were no significant differences in auditory threshold shifts observed between NE18 and E18 animals (Figure 2G).

### Wave Amplitudes

**FIGURE 3:**
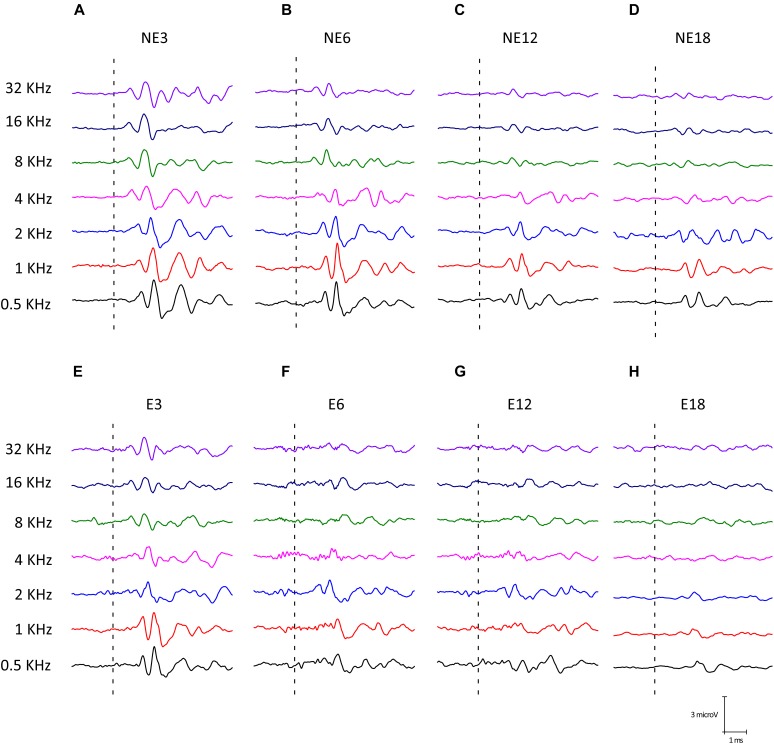
Examples of ABR waveforms from NE **(A–D)** and E **(E–H)** rats at all ages and frequencies evaluated. The NE3 **(A)**, NE6 **(B)**, and E3 © animals, previous any noise exposure, showed typical waveform patterns which consisted of 4 to 5 evoked waves after stimulus onset. At 12 **(C)** and 18 **(D)** months of age, NE rats had an age-related decrease in wave amplitudes at all frequencies. However, in the exposed animals such a reduction was already apparent at 6 months **(F)** and it was more evident at 12 **(G)** and 18 **(H)** months of age for the higher frequencies when compared to NE rats.

Illustrative examples of ABR recordings from NE (Figure 3A–D) and E (Figure 3E–H) rats at the different groups of age are depicted in Figure 3. The typical waveform pattern described elsewhere for Wistar rats (Overbeck and Church, 1992; Church et al., 2010, 2012b,a; Alvarado et al., 2012, 2014, 2018) was observed in NE3 (Figure 3A) and NE6 (Figure 3B), as well as in E3 (Figure 3E) animals. Consistent with previous studies (Alvarado et al., 2014, 2018), an age-related decrease in wave amplitudes at all frequencies evaluated was found at 12 (Figure 3C) and (Figure 3D) 18 months of age, both in NE and E groups. However, in rats in the E group, the decrease in wave amplitudes was already present at 6 months of age (E6) (Figure 3F) and was more evident at the highest frequencies in E12 (Figure 3G) and E18 (Figure 3H) animals.

**FIGURE 4:**
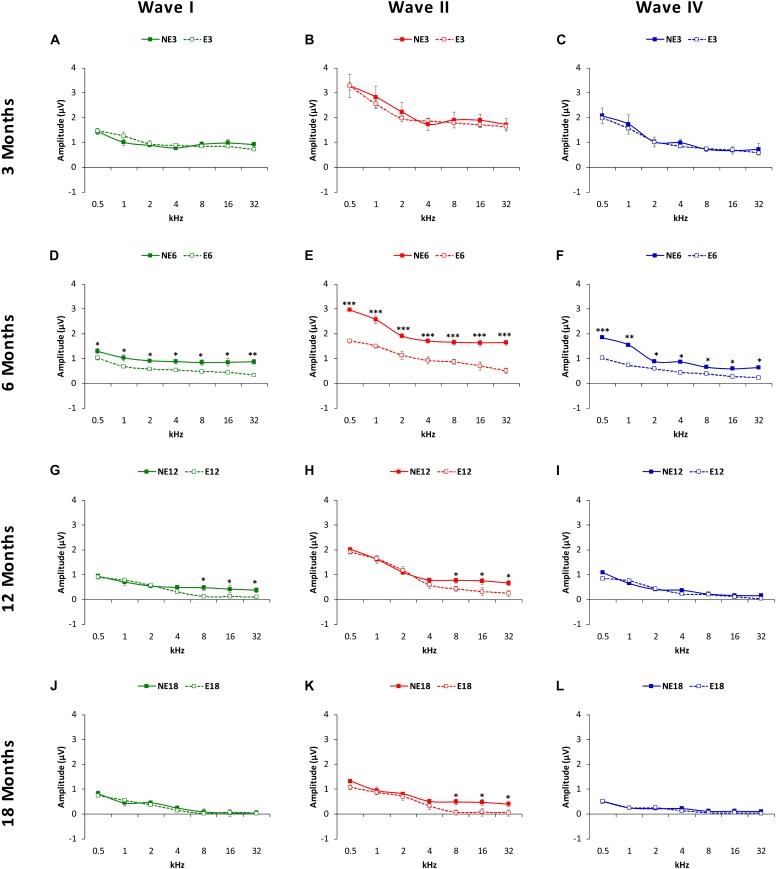
Line graphs illustrating wave amplitudes (in μV), as a function of the frequencies evaluated in NE (solid lines) and E (dashed lines) animals at 3, 6, 8, and 18 months of age. At 3 months **(A–C)**, previous any noise exposure, the mean values of waves I, II and IV (the largest in the ABRs) were similar in both groups, while in 6-month-old rats **(D–F)**, the mean amplitudes of all waves in the exposed animals at all frequencies were significantly reduced when compared to non-exposed rats. At 12 months **(G–I)**, while there was a decrease in the mean amplitudes of all waves in both exposed and non-exposed rats, waves I **(G)** and II **(H)** at the highest frequencies **(H)** in the E group were still smaller than those observed in NE animals. No differences were observed in wave IV **(I)**. At 18 months, whereas no differences were observed between groups in the waves I **(J)** and IV **(L)**, the mean values in wave II in the E group remained reduced at the highest frequencies **(K)**. ^∗^*p* < 0.05, ^∗∗^*p* < 0.01, ^∗∗∗^*p* < 0.001.

Evaluation of mean amplitude values of the largest waves in the ABRs (see MATERIALS AND METHODS, Figure 4) with ANOVA, indicated that there was a significant effect of noise overstimulation and age on the amplitudes of waves I (*F*_(3,__258)_ = 2fb.64, *p* < 0.05], II [F_(3,__258)_ = 3.19, *p* < 0.01] and IV [F(_3,__258)_ = 2.54, *p* < 0.05], already evident as early as 6 months in the exposed animals. In other words, at 3 months of age (Figure 4), the amplitudes of wave I (Figure 4A, green), II (Figure 4B, red), and IV (Figure 4C, blue) were similar between NE (solid lines) and E (dashed lines) animals. However, in groups at older ages, comparison between exposed and non-exposed animals at different time-points demonstrated that at 6 months, the mean amplitudes in the NE rats were still similar to those observed in 3-month-old rats whereas in the E animals there was a significant decrease in the magnitude of all waves at all frequencies studied (Figure 4D–F). In 12-month-old rats, the mean values at 8, 16, and 32 kHz for waves I (Figure 4G) and II (Figure 4H), but not for wave IV (Figure 4I), were significantly lower in the E animals as compared to NE animals. By 18 months (Figure 4J–L), the only significant difference that persisted was that observed in wave II at 8, 16, and 32 kHz (Figure 4K).

**FIGURE 5:**
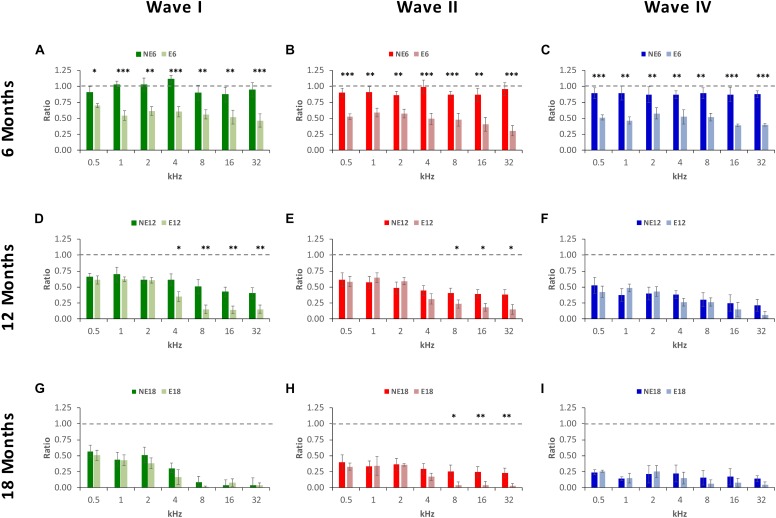
Bar graphs illustrating wave amplitude ratios in noise-exposed animals at 6, 12, and 18 months of age relative to the control condition. In 6-month-old exposed animals, **(A–C)**, wave amplitude ratios for all waves and at all frequencies were significantly smaller when compared to the non-exposed rats. At 12 months, while ratios decreased in both NE and E groups, in E animals the mean values were still significantly reduced at the highest frequencies for waves I **(D)** and II **(E)**, but not for wave IV **(F)**. In the oldest animals **(G–I)**, the mean values remained reduced in both groups and significant differences were observed only in wave II **(H)** at 8, 16, and 32 kHz. ^∗^*p* < 0.05, ^∗∗^*p* < 0.01, ^∗∗∗^*p* < 0.001.

ANOVA of the normalized waveform amplitude using the wave amplitude ratio (see MATERIALS AND METHODS), confirmed the above mentioned differences between exposed and non-exposed groups, suggesting that there was a significant effect of noise overstimulation and age on the amplitude ratio of waves I [*F*(_2,__190)_ = 3.39, *p* < 0.05], II [*F*_(2,__190)_ = 5.75, *p* < 0.01] and IV [*F*(_2,__190)_ = 6.69, *p* < 0.01]. At 6 months of age, in NE rats, ratios ranged from 0.87 to 1.11 in wave I (Figure 5A), from 0.87 to 0.99 in wave II (Figure 5B), and from 0.86 to 0.89 in wave IV (Figure 5C). These ratios were significantly lower in E rats for all waves and at all frequencies evaluated, not exceeding values of 0.70, 0.59, and 0.58 in waves I (Figure 5A), II (Figure 5B), and IV (Figure 5C), respectively. At 12 months of age, in the NE animals, ratios ranged from 0.41 to 0.70 in wave I (Figure 5D), from 0.38 to 0.61 in wave II (Figure 5E), and from 0.21 to 0.52 in wave IV (Figure 5F); whereas in the E animals, the ratios ranged from 0.14 to 0.62 in wave I (Figure 5D), from 0.15 to 0.65 in wave II (Figure 5E), and from 0.07 to 0.49 in wave IV (Figure 5F). These ratios were statistically significantly lower in wave I at 4, 8, 16, and 32 kHz, and in wave II at 8, 16, and 32 kHz. Finally, in 18-month-old animals, the wave amplitude ratios in the NE rats were from 0.04 to 0.51 in wave I (Figure 5G), from 0.23 to 0.40 in wave II (Figure 5H) and from 0.14 to 0.24 in wave IV (Figure 5I); however, in the E rats, these values ranged from 0.01 to 0.51 in wave I (Figure 5G), from 0.03 to 0.36 in wave II (Figure 5H), and from 0.05 to 0.26 in wave IV (Figure 5I), with significant differences only in wave II at 8, 16, and 32 kHz.

### Wave Latencies

**FIGURE 6:**
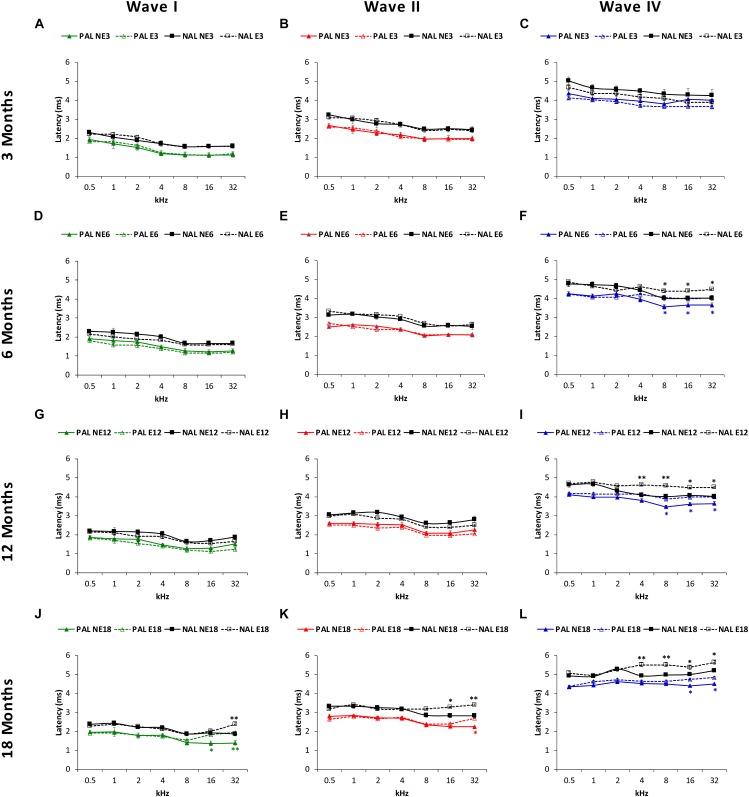
Line graphs depicting the absolute positive (colored lines) and negative (black lines) latency times (ms) of waves I, II, and IV, as a function of frequency in NE and E rats. At 3 months **(A–C)** in both NE and E animals, previous any noise exposure, the mean values for the absolute latencies in waves I, II, and IV were similar to those previously reported. At 6 and 12 months in the exposed rats these values were significantly longer at the highest frequencies for wave IV **(F,I)**, but not for wave I **(D,G)** or II **(E,H)**. In 18-month-old rats, a significant lengthening of latency times was detected in the exposed animals for waves I **(J)**, II **(K)**, and IV **(L)** also at the highest frequencies. PAL, Positive Absolute Latencies; NAL, Negative Absolute Latencies, ^∗^*p* < 0.05, ^∗∗^*p* < 0.01.

The mean values observed in NE3 and E3 rats, in both the positive and negative (Figure 6A–C) absolute latencies, were consistent with those previously reported (Jamesdaniel et al., 2008; Church et al., 2010, 2012b; Alvarado et al., 2012, 2014, 2016, 2018; Fuentes-Santamaría et al., 2012, 2013, 2014; Pilati et al., 2012; Melgar-Rojas et al., 2015a). However, in the older animals, ANOVA demonstrated a significant effect of noise overstimulation and age on the positive and negative absolute latencies of wave I [*F*(_3,__258)_ = 2.34, *p* < 0.05, for the positive latency; *F*_(3,__258)_ = 2.37, *p* < 0.05, for the negative latency], II [*F*(_3,__258)_ = 2.36, *p* < 0.05, for the positive latency; *F*(_3,__258)_ = 2.38, *p* < 0.05, for the negative latency] and IV [*F*_(3,__258)_ = 2.79, *p* < 0.05, for the positive latency; *F*(_3,__258)_ = 3.25, *p* < 0.05, for the negative latency]. In all cases, there were longer latency times at higher frequencies in the exposed rats as compared to non-exposed animals. Nevertheless, the effect was more evident in wave IV than in waves I and II. Accordingly, for the absolute positive latency, significantly longer times in wave IV were present in E6 (8, 16, and 32 kHz), E12 (8, 16, and 32 kHz) and E18 (16 and 32 kHz) groups (blue lines in Figure 6F,I,L), and also in the E18 group in wave 1 at 16 and 32 kHz (green lines in Figure 6D,G,J) and in the E18 group in wave II at 32 kHz (red lines in Figure 6E,H,K). As far as absolute negative latencies are concerned, our data demonstrate that wave IV showed statistically significantly longer times in the E6 (8, 16, and 32 kHz), E12 (4, 8, 16, and 32 kHz) and E18 (4, 8, 16, and 32 kHz) groups (black lines in Figure 6F,I,L), whereas in waves I (black lines in Figure 6D,G,J) and II (black lines in Figure 6E,H,K), significantly longer latencies were observed in the E18 group at 32 kHz, and at 16 and 32 kHz, respectively.

**FIGURE 7:**
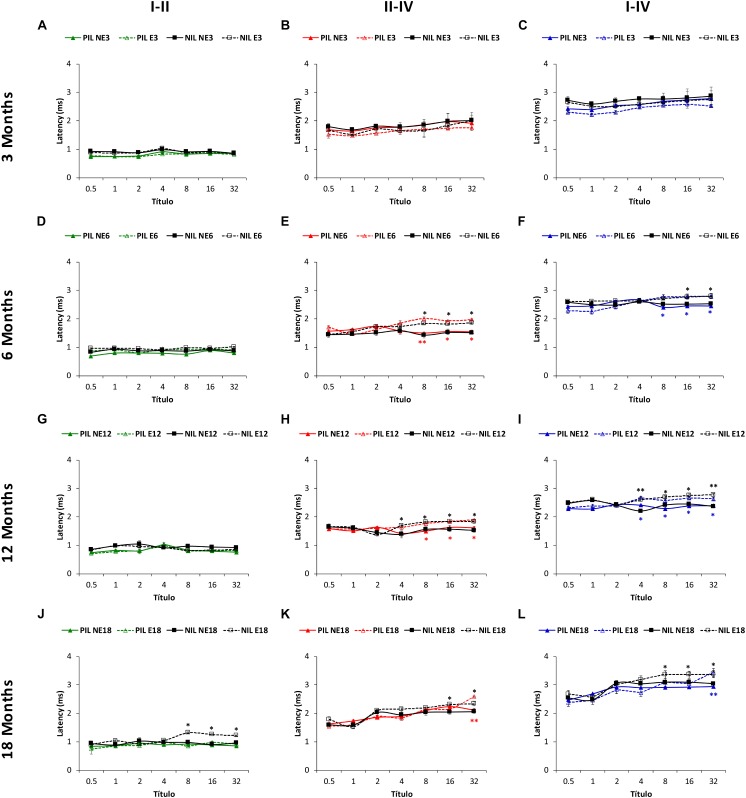
Line graphs illustrating the interpeak positive (colored lines) and negative (black lines) latency times (ms) plotted as a function of frequency in non-exposed and exposed rats. In the 3-month-old group, previous any noise exposure, there were no differences between E and NE groups in any of the interpeak positive latencies evaluated **(A–C)**. However, there was a significant effect of sound stimulation and age on the interpeak positive latency times at 6 **(D–F)**, 12 **(G–I)**, and 18 **(J–L)** months. Accordingly, in the I-II interpeak positive latency times whereas no differences were observed between NE and E rats, longer negative latencies in the exposed animals were detected at the highest frequencies in 18-month-old **(D–J)** animals. In the II-IV **(E,H,K)** and I-IV **(F,I,L)** interpeak latencies, longer times were detected in the exposed animals at the highest frequencies at 6, 12, and 18 months when compared to non-exposed rats. PIL, Positive Interpeak Latencies; NAL, Negative Interpeak Latencies, ^∗^*p* < 0.05, ^∗∗^*p* < 0.01.

Similar to the absolute latencies, the mean values in NE and E rats at 3 months of age for the positive as well as for the negative (Figure 7A–C) interpeak latencies, were consistent with those observed previously (Jamesdaniel et al., 2008; Church et al., 2010, 2012b; Alvarado et al., 2012, 2014, 2016, 2018; Fuentes-Santamaría et al., 2012, 2013, 2014; Pilati et al., 2012; Melgar-Rojas et al., 2015a). ANOVA also revealed a significant effect of noise overstimulation and age on the positive interpeak latencies II-IV [*F*(_3,__258)_ = 2.58, *p* < 0.05] and I–IV [*F*_(3,__258)_ = 2.73, *p* < 0.05], and in the negative interpeak latencies I-II [*F*_(3,__258)_ = 7.02, *p* < 0.001], II-IV [*F*_(3,__258)_ = 3.37, *p* < 0.05] and I-IV [*F*(_3,__258)_ = 5.52, *p* < 0.01]. In the positive interpeak latencies, the Scheffé *post hoc* test indicated that, when compared to the non-exposed rats, older exposed animals had significantly longer interpeak times between waves II and IV in the E6 (8, 16, 32 kHz), E12 (8, 16, 32 kHz) and E18 (32 kHz) groups (red lines in Figure 7E,H,K). Also, statistically significantly longer interpeak times were detected in the exposed rats, between waves I and IV in the E6 (8, 16, 32 kHz), E12 (4, 8, 16, 32 kHz) and E18 (32 kHz) groups (blue lines in Figure 7F,I,L). No significant differences were observed in the interpeak times between waves I and II (green lines in Figure 7D,G,J). For the negative interpeak latencies, the Scheffé *post hoc* test also demonstrated longer interpeak times in the exposed rats. Actually, the interpeak times between waves I and II, did not differ between 6 (black lines in Figure 7D) and 12-month old (black lines in Figure 7G) rats. However, the mean values were statistically significantly longer in E18 (8, 16, 32 kHz) animals (black lines in Figure 7J). In the interpeak times between waves II and IV, significantly longer latencies were observed at 6 (8, 16, 32 kHz), 12 (4, 8, 16, 32 kHz) and 18 (16, 32 kHz) months of age (black lines in Figure 7E,H,K). Finally, in the interpeak latencies between waves I and IV, significant differences were found at 6 (16, 32 kHz), 12 (4, 8, 16, 32 kHz) and 18 (8, 16, 32 kHz) moths of age (black lines in Figure 7F,I,L).

## Discussion

The findings in the present study suggest that repeated short-duration loud sound overstimulation accelerates the time-course of ARHL in an animal model of auditory aging. Alterations in auditory function were present as early as 6 months of age following sound exposure and were characterized by significant increases in auditory thresholds, decreases in waveform amplitudes and longer latency times. All these ABR parameters in the exposed animals showed variations in mean values when compared to their corresponding age-matched non-exposed rats. They were, however, comparable to those found in older non-exposed animals. In this regard, threshold shifts, reduced wave amplitudes and longer latency times in the E6 and E12 groups, all corresponded with those typically observed in the NE12 and NE18 animals, respectively, suggesting that changes in auditory function related to ARHL are accelerated after prolonged repetitive sub-damaging noise exposure. To our knowledge, this is the first study characterizing the effects of a continuous long-term noise exposure in an animal model of ARHL. In summary, these results support that daily exposure to short-duration loud sounds causes modifications in ABR recordings indicative of early hearing loss. Such noise-induced functional abnormalities in the usual auditory threshold and wave patterns are similar to those seen in aged subjects as a consequence of ARHL.

Noise exposure and aging have long been associated with hearing impairment, either independently or coexisting (World Health Organization, 1997, 2017a,b; Clark and Bohne, 1999; Goelzer, 2001; Chisolm et al., 2003; Gordon-Salant, 2005; Daniel, 2007; Bielefeld et al., 2010; Basner et al., 2014). Although, the nature of interactions between noise and auditory aging is still a matter of debate, the prevailing view is that noise modifies the onset and/or progression of ARHL (Turner and Willott, 1998; Willott and Turner, 1999; Tanaka et al., 2009; Bielefeld et al., 2010; Fetoni et al., 2011). Nevertheless, it is worth noting that the outcome of noise exposure during aging will depend on the properties of the sound, such as intensity and duration. For instance, mice exposed to a non-traumatic 70 dB SPL broad-band noise 12 h every night (augmented acoustic environment) for a long time show an improvement in their hearing, reflected in reduced threshold shift, enhancement of the central auditory function and delayed onset of ARHL when compared to age-matched non-exposed mice (Turner and Willott, 1998; Willott and Turner, 1999; Willott et al., 2000; Tanaka et al., 2009; Bielefeld et al., 2010). It has been proposed that the mechanisms derived from the augmented acoustic environment that participate in ameliorating ARHL could be similar to those observed during auditory priming, which is a phenomenon that helps to protect the auditory system from future exposures to damaging noise (Canlon et al., 1988; Subramaniam et al., 1991; Canlon, 1997; Pukkila et al., 1997; Brown et al., 1998; Attanasio et al., 1999; Hamernik and Ahroon, 1999; Kujawa and Liberman, 1999; Yamasoba et al., 1999; Niu and Canlon, 2002; Gourévitch et al., 2009; Alvarado et al., 2016).

On the contrary, the consequences of the exposure to higher intensity sounds during aging are the acceleration and / or exacerbation of ARHL (Gates and Mills, 2005; Kujawa and Liberman, 2006; Bielefeld et al., 2010; Fetoni et al., 2011; Fernandez et al., 2015). Consistent with these observations, the present findings demonstrate that repeated exposure to a short duration loud sound (1 h/110 dB SPL) during a long time period induces changes in ABR recording parameters suggestive of an accelerated onset of ARHL. Under normal conditions, ARHL onset in Wistar rats, occurs around 12 months of age (Alvarado et al., 2014). However, following a repeated short-duration loud sound exposure, it is accelerated in such a way that it is already present at 6 months. As demonstrated in this study, the mean values of auditory thresholds and the threshold shifts in the exposed animals at 6 and 12 months of age showed PTS with threshold shifts between 20 and 40 dB, similar to those seen in the non-exposed control rats at 12 and 18 months, respectively and to those reported previously for Wistar rats at the same ages (Alvarado et al., 2014). At present, there are very few reports evaluating the long-term effects of a single exposure to high level noise stimulation. In this regard, studies in CBA/CaJ mice exposed to 8–16 kHz, 100 dB SPL for 2 h have demonstrated functional and histopathological alterations during aging such as increases in auditory thresholds and threshold shifts, loss of spiral ganglion neurons and degeneration of synapses between inner hair cells and cochlear nerve terminals (“cochlear synaptopathy”). All these are features compatible with ARHL that are not present in age-matched unexposed mice (Kujawa and Liberman, 2006; Sergeyenko et al., 2013; Fernandez et al., 2015). Thus, it may be plausible that, in addition to i4ncreased oxidative stress and reduced cochlear blood flow, the above-mentioned abnormalities may also account for the alterations in auditory thresholds described here for the Wistar strain. In this study, Wistar rats were used as animal models of ARHL as they show a progressive age-related decline in the auditory thresholds that is more pronounced at higher frequencies (Alvarado et al., 2014; Möhrle et al., 2016), which represents a functional hallmark of presbycusis in humans and ARHL in other animal models (Boettcher, 2002; Syka, 2002; Bielefeld et al., 2010; Huang and Tang, 2010; Fetoni et al., 2011). The Wistar strain also shares many of the peripheral and central alterations of ARHL that have been previously described in other animal models including the F344 rats, a frequently used strain for the evaluation of ARHL (Popelar et al., 2006; Bielefeld et al., 2008, 2010; Syka, 2010). However, as an animal model of ARHL, Wistar rats have several advantages over other rat strains such as Long-Evans, which show only subtle modifications in auditory function in the oldest animals, and over F344, since this strain exhibits a very high incidence of spontaneous tumors and degenerative diseases that may interfere with the interpretation of the results (Popelar et al., 2006; Syka, 2010; Rybalko et al., 2012). Therefore, the Wistar strain represents an appropriate model suitable for long-term experimental studies on ARHL.

Our results also demonstrate that modifications observed in the noise-exposed Wistar rats were not restricted to auditory thresholds, as there was a significant reduction in the magnitude of the responses, reflected in a decrease in wave amplitudes and lengthened latency times, both absolute and interpeak. Such age-related modifications have been described elsewhere for 12 and 18-month-old Wistar rats (Alvarado et al., 2014). In this regard, our previous data suggest that these alterations in evoked responses could be due to several factors such as decreased excitatory cochlear inputs, impaired synaptic afferents in auditory nuclei or impaired neurotransmission along the auditory pathway (Popelar et al., 2006; Alvarado et al., 2014), supporting the idea that in addition to “peripheral presbycusis,” there is also a “central presbycusis” (Gates and Mills, 2005; Humes et al., 2012) in the Wistar strain. In the noise-exposed rats, reduction in wave amplitudes was already apparent at 6 months, in all waves and at all frequencies evaluated. At 12 months, this reduction persisted in wave I and II at the highest frequencies and by 18 months, it remained at the highest frequencies in wave II. The longest absolute latency times were observed mostly for wave IV at 6, 12, and 18 months at the highest frequencies evaluated, while lengthening of interpeak latencies was detected between waves II-IV and I-IV. These results suggest that the interaction between noise and aging not only takes place in the peripheral auditory system but also in the central auditory system, contributing to accelerating presbycusis.

The findings in the present study, together with previous reports, support the notion that the interplay between loud, but sub-damaging noise and age may contribute to modify the onset and/or progression of presbycusis. This fact is important for two main reasons: (1) improvement in life quality and better health care systems have led to longer life expectancy and consequently, to a higher risk of suffering ARHL; (2) exposure to greater levels of loud sound or excessive noise is increasing dramatically in modern societies (World Health Organization, 1997). In this regard, while occupational noise tends to reduction in developed countries, it still remains a major public health problem worldwide (World Health Organization, 1997, 2006, 2018;  Śliwińska-Kowalska and Davis, 2012;  Śliwińska-Kowalska and Davis, 2017). In addition, there is a growing concern about the increasing exposure to noise related to social interactions, mainly recreational and leisure noise. Leisure activities could generate broadband sounds with sound levels that could reach and even exceed 110 dB(A) (World Health Organization, 1997, 2006, 2018;  Śliwińska-Kowalska and Davis, 2012;  Śliwińska-Kowalska and Davis, 2017). Within these activities, the population groups at risk are mostly children, teenagers and young adults, especially due to the use of personal portable audio devices such as the smartphones and MP3 (Daniel, 2007; European Commission, 2008; Kim et al., 2009;  Śliwińska-Kowalska and Davis, 2012; Basner et al., 2014;  Śliwińska-Kowalska and Davis, 2017). It has been estimated that the mean time exposure to music devices ranges from 1 to 14 h per week (European Commission, 2008;  Śliwińska-Kowalska and Davis, 2012) which represents a long-term potential health risk. It is worth noting that there seems to be a “critical period” in which there is a greater interaction between noise and age, and that such effect diminishes with aging, being older subjects less vulnerable to noise (Henry, 1982; Kujawa and Liberman, 2006). Hence, it could be assumed that the young population exposed voluntarily to loud music for 1 h every day for long time periods are at risk of developing premature presbycusis with all its consequences. In support of this argument, our results demonstrate that differences between noise-exposed and non-exposed rats were maximal at 6 months of age decreasing steadily until 18 months, suggesting a reduced effect of noise exposure and age on hearing. In conclusion, we provide evidence of a noise/age synergistic interaction in response to a repeated 1 h exposure to a loud sound for a prolonged time period which may contribute to accelerate the onset and progression of ARHL.

## Author Contributions

All authors had full access to all the data in the study and took responsibility for the integrity of the data and the accuracy of the data analysis. JA and VF-S: studied concept and design. JA, VF-S, and MG-U: were responsible for acquisition of data. JA and VF-S: were responsible for statistical analysis and interpretation of data. VF-S and JA: drafted the manuscript. JA, VF-S, and JJ: critically revised the manuscript for important intellectual content. JJ: obtained funding.

## Conflict of Interest Statement

The authors declare that the research was conducted in the absence of any commercial or financial relationships that could be construed as a potential conflict of interest.

## References

[B1] Alvarado J. C. Fuentes-Santamaría V. Gabaldón-Ull M. C. Blanco J. L. Juiz J. M. (2014). Wistar rats: a forgotten model of age-related hearing loss. *Front. Aging Neurosci.* 6:29. 10.3389/fnagi.2014.00029 24634657 PMC3942650

[B2] Alvarado J. C. Fuentes-Santamaría V. Gabaldón-Ull M. C. Jareño-Flores T. Miller J. M. Juiz J. M. (2016). Noise-induced “Toughening” effect in wistar rats: enhanced auditory brainstem responses are related to calretinin and nitric oxide synthase upregulation. *Front. Neuroanat.* 10:19 10.3389/fnana.2016.00019 PMC4815363 27065815

[B3] Alvarado J. C. Fuentes-Santamaría V. Gabaldón-Ull M. C. Juiz J. M. (2018). An oral combination of vitamins A, C, E, and Mg++ improves auditory thresholds in age-related hearing loss. *Front. Neurosci.* 12:527. 10.3389/fnins.2018.00527 30108480 PMC6079267

[B4] Alvarado J. C. Fuentes-Santamaría V. Jareño-Flores T. Blanco J. L. Juiz J. M. (2012). Normal variations in the morphology of auditory brainstem response (ABR) waveforms: a study in wistar rats. *Neurosci. Res.* 73 302–311. 10.1016/j.neures.2012.05.001 22595234

[B5] Alvarado J. C. Fuentes-Santamaría V. Melgar-Rojas P. Valero M. L. Gabaldón-Ull M. C. Miller J. M. (2015). Synergistic effects of free radical scavengers and cochlear vasodilators: a new otoprotective strategy for age-related hearing loss. *Front. Aging Neurosci.* 7:86. 10.3389/fnagi.2015.00086 26029103 PMC4432684

[B6] Attanasio G. Barbara M. Buongiorno G. Cordier A. Mafera B. Piccoli F. (1999). Protective effect of the cochlear efferent system during noise exposure. *Ann. N. Y. Acad. Sci.* 884 361–367. 10.1111/j.1749-6632.1999.tb08654.x 10842606

[B7] Basner M. Babisch W. Davis A. Brink M. Clark C. Janssen S. (2014). Auditory and non-auditory effects of noise on health. *Lancet* 383 1325–1332. 10.1016/S0140-6736(13)61613-X 24183105 PMC3988259

[B8] Berglund B. Lindvall T. Schwela D. H. World Health Organization (1999). *Guidelines for Community Noise.* Geneva: World Health Organization.

[B9] Bielefeld E. C. Coling D. Chen G.-D. Li M. Tanaka C. Hu B.-H. (2008). Age-related hearing loss in the Fischer 344/NHsd rat substrain. *Hear. Res.* 241 26–33. 10.1016/j.heares.2008.04.006 18508213 PMC2556048

[B10] Bielefeld E. C. Tanaka C. Chen G. Henderson D. (2010). Age-related hearing loss: is it a preventable condition? *Hear. Res.* 264 98–107. 10.1016/j.heares.2009.09.001 19735708 PMC2868117

[B11] Boettcher F. A. (2002). Presbyacusis and the auditory brainstem response. *J. Speech Lang. Hear. Res.* 45 1249–1261. 10.1044/1092-4388(2002/100) 12546491

[B12] Boettcher F. A. Mills J. H. Swerdloff J. L. Holley B. L. (1996). Auditory evoked potentials in aged gerbils: responses elicited by noises separated by a silent gap. *Hear. Res.* 102 167–178. 10.1016/S0378-5955(96)90016-7 8951460

[B13] Brown M. C. Kujawa S. G. Liberman M. C. (1998). Single olivocochlear neurons in the guinea pig. II. Response plasticity due to noise conditioning. *J. Neurophysiol.* 79 3088–3097. 10.1152/jn.1998.79.6.3088 9636110

[B14] Canlon B. (1997). Protection against noise trauma by sound conditioning. *Ear Nose Throat J.* 76 248–250, 253–255. 9127524

[B15] Canlon B. Borg E. Flock Å. (1988). Protection against noise trauma by pre-exposure to a low level acoustic stimulus. *Hear. Res.* 34 197–200. 10.1016/0378-5955(88)90107-4 3170362

[B16] Cediel R. Riquelme R. Contreras J. Díaz A. Varela-Nieto I. (2006). Sensorineural hearing loss in insulin-like growth factor I-null mice: a new model of human deafness: hearing loss in Igf-1-mutant mice. *Eur. J. Neurosci.* 23 587–590. 10.1111/j.1460-9568.2005.04584.x 16420467

[B17] Chen T. J. Chen S. S. (1991). Generator study of brainstem auditory evoked potentials by a radiofrequency lesion method in rats. *Exp. Brain Res.* 85 537–542. 10.1007/BF00231737 1915709

[B18] Chiappa K. H. Gladstone K. J. Young R. R. (1979). Brain stem auditory evoked responses: studies of waveform variations in 50 normal human subjects. *Arch. Neurol.* 36 81–87. 10.1001/archneur.1979.00500380051005 420627

[B19] Chisolm T. H. Willott J. F. Lister J. J. (2003). The aging auditory system: anatomic and physiologic changes and implications for rehabilitation. *Int. J. Audiol.* 42(Suppl. 2), S3–S10. 10.3109/14992020309074637 12918622

[B20] Church M. W. Adams B. R. Anumba J. I. Jackson D. A. Kruger M. L. Jen K.-L. C. (2012a). Repeated antenatal corticosteroid treatments adversely affect neural transmission time and auditory thresholds in laboratory rats. *Neurotoxicol. Teratol.* 34 196–205. 10.1016/j.ntt.2011.09.004 21963399 PMC3268869

[B21] Church M. W. Hotra J. W. Holmes P. A. Anumba J. I. Jackson D. A. Adams B. R. (2012b). Auditory brainstem response (ABR) abnormalities across the life span of rats prenatally exposed to alcohol. *Alcohol. Clin. Exp. Res.* 36 83–96. 10.1111/j.1530-0277.2011.01594.x 21815896 PMC3210930

[B22] Church M. W. Jen K.-L. C. Anumba J. I. Jackson D. A. Adams B. R. Hotra J. W. (2010). Excess omega-3 fatty acid consumption by mothers during pregnancy and lactation caused shorter life span and abnormal ABRs in old adult offspring. *Neurotoxicol. Teratol.* 32 171–181. 10.1016/j.ntt.2009.09.006 19818397 PMC2839050

[B23] Clark W. W. Bohne B. A. (1999). Effects of noise on hearing. *JAMA* 281 1658–1659. 10.1001/jama.281.17.1658 10235164

[B24] Daniel E. (2007). Noise and hearing loss: a review. *J. Sch. Health* 77 225–231. 10.1111/j.1746-1561.2007.00197.x 17430434

[B25] European Commission (2008). *Potential Health Risks of Exposure to Noise from Personal Music Players and Mobile Phones Including a Music Playing Function.* Brussels: European Commission.

[B26] Fernandez K. A. Jeffers P. W. C. Lall K. Liberman M. C. Kujawa S. G. (2015). Aging after noise exposure: acceleration of cochlear synaptopathy in “Recovered” ears. *J. Neurosci.* 35 7509–7520. 10.1523/JNEUROSCI.5138-14.2015 25972177 PMC4429155

[B27] Fetoni A. R. Picciotti P. M. Paludetti G. Troiani D. (2011). Pathogenesis of presbycusis in animal models: a review. *Exp. Gerontol.* 46 413–425. 10.1016/j.exger.2010.12.003 21211561

[B28] Fuentes-Santamaría V. Alvarado J. C. Gabaldón-Ull M. C. Juiz J. M. (2013). Upregulation of insulin-like growth factor and interleukin 1β occurs in neurons but not in glial cells in the cochlear nucleus following cochlear ablation: upregulation of IGF-1 and IL-1β in cochlear nucleus. *J. Comp. Neurol.* 521 3478–3499. 10.1002/cne.23362 23681983

[B29] Fuentes-Santamaría V. Alvarado J. C. Juiz J. M. (2012). Long-term interaction between microglial cells and cochlear nucleus neurons after bilateral cochlear ablation. *J. Comp. Neurol.* 520 2974–2990. 10.1002/cne.23088 22351306

[B30] Fuentes-Santamaría V. Alvarado J. C. López-Muñoz D. F. Melgar-Rojas P. Gabaldón-Ull M. C. Juiz J. M. (2014). Glia-related mechanisms in the anteroventral cochlear nucleus of the adult rat in response to unilateral conductive hearing loss. *Front. Neurosci.* 8:319. 10.3389/fnins.2014.00319 25352772 PMC4195288

[B31] Fujimoto C. Yamasoba T. (2014). Oxidative stresses and mitochondrial dysfunction in age-related hearing loss. *Oxid. Med. Cell. Longev.* 2014:582849. 10.1155/2014/582849 25110550 PMC4106174

[B32] Garcia-Pino E. Caminos E. Juiz J. M. (2009). KCNQ5 reaches synaptic endings in the auditory brainstem at hearing onset and targeting maintenance is activity-dependent. *J. Comp. Neurol.* 518 1301–1314. 10.1002/cne.22276 20151361

[B33] Gates G. A. Mills J. H. (2005). Presbycusis. *Lancet* 366 1111–1120. 10.1016/S0140-6736(05)67423-5 16182900

[B34] Goelzer B. (ed.). (2001). *Occupational Exposure to Noise: Evaluation, Prevention and Control.* Bremerhaven: Wirtschaftsverl.

[B35] Gopinath B. Rochtchina E. Wang J. J. Schneider J. Leeder S. R. Mitchell P. (2009). Prevalence of age-related hearing loss in older adults: blue mountains study. *Arch. Intern. Med.* 169 415–416. 10.1001/archinternmed.2008.597 19237727

[B36] Gordon-Salant S. (2005). Hearing loss and aging: new research findings and clinical implications. *J. Rehabil. Res. Dev.* 42(4 Suppl. 2), 9–24. 10.1682/JRRD.2005.01.0006 16470462

[B37] Gourévitch B. Doisy T. Avillac M. Edeline J.-M. (2009). Follow-up of latency and threshold shifts of auditory brainstem responses after single and interrupted acoustic trauma in guinea pig. *Brain Res.* 1304 66–79. 10.1016/j.brainres.2009.09.041 19766602

[B38] Hamernik R. P. Ahroon W. A. (1999). Sound-induced priming of the chinchilla auditory system. *Hear. Res.* 137 127–136. 10.1016/S0378-5955(99)00131-8 10545640

[B39] Henderson D. Bielefeld E. C. Harris K. C. Hu B. H. (2006). The role of oxidative stress in noise-induced hearing loss. *Ear Hear.* 27 1–19. 10.1097/01.aud.0000191942.36672.f3 16446561

[B40] Henry K. R. (1982). Age-related changes in sensitivity of the postpubertal ear to acoustic trauma. *Hear. Res.* 8 285–294. 10.1016/0378-5955(82)90020-X 7153183

[B41] Huang Q. Tang J. (2010). Age-related hearing loss or presbycusis. *Eur. Arch. Otorhinolaryngol.* 267 1179–1191. 10.1007/s00405-010-1270-7 20464410

[B42] Humes L. E. Dubno J. R. Gordon-Salant S. Lister J. J. Cacace A. T. Cruickshanks K. J. (2012). Central presbycusis: a review and evaluation of the evidence. *J. Am. Acad. Audiol.* 23 635–666. 10.3766/jaaa.23.8.5 22967738 PMC5898229

[B43] Jamesdaniel S. Ding D. Kermany M. H. Davidson B. A. Knight P. R. III Salvi R. (2008). Proteomic analysis of the balance between survival and cell death responses in cisplatin-mediated ototoxicity. *J. Proteome Res.* 7 3516–3524. 10.1021/pr8002479 18578524 PMC2570323

[B44] Kim M. G. Hong S. M. Shim H. J. Kim Y. D. Cha C. I. Yeo S. G. (2009). Hearing threshold of Korean adolescents associated with the use of personal music players. *Yonsei Med. J.* 50 771–776. 10.3349/ymj.2009.50.6.771 20046416 PMC2796402

[B45] Kujawa S. G. Liberman M. C. (1999). Long-term sound conditioning enhances cochlear sensitivity. *J. Neurophysiol.* 82 863–873. 10.1152/jn.1999.82.2.863 10444683

[B46] Kujawa S. G. Liberman M. C. (2006). Acceleration of age-related hearing loss by early noise exposure: evidence of a misspent youth. *J. Neurosci.* 26 2115–2123. 10.1523/JNEUROSCI.4985-05.2006 16481444 PMC1855187

[B47] Le Prell C. G. Hughes L. Miller J. M. (2007a). Free radical scavengers vitamins A, C, and E plus magnesium reduce noise trauma. *Free Radic. Biol. Med.* 42 1454–1463. 10.1016/j.freeradbiomed.2007.02.008 17395018 PMC1950331

[B48] Le Prell C. G. Yamashita D. Minami S. B. Yamasoba T. Miller J. M. (2007b). Mechanisms of noise-induced hearing loss indicate multiple methods of prevention. *Hear. Res.* 226 22–43. 10.1016/j.heares.2006.10.006 17141991 PMC1995566

[B49] Lee K.-Y. (2013). Pathophysiology of age-related hearing loss (Peripheral and Central). *Korean J. Audiol.* 17 45–49. 10.7874/kja.2013.17.2.45 24653905 PMC3936539

[B50] Lin F. R. Thorpe R. Gordon-Salant S. Ferrucci L. (2011). Hearing loss prevalence and risk factors among older adults in the united states. *J. Gerontol. A Biol. Sci. Med. Sci.* 66A, 582–590. 10.1093/gerona/glr002 21357188 PMC3074958

[B51] Mathers C. Fat D. M. Boerma J. T. World Health Organization (eds). (2008). *The Global Burden of Disease: 2004 Update.* Geneva: World Health Organization 10.1016/B978-012373960-5.00335-X

[B52] Mathers C. Smith A. Concha M. (2000). Global burden of hearing loss in the year 2000. *Glob. Burd. Dis.* 18 1–30.

[B53] Melgar-Rojas P. Alvarado J. C. Fuentes-Santamaría V. Gabaldón-Ull M. C. Juiz J. M. (2015a). Validation of reference genes for RT–qPCR analysis in noise–induced hearing loss: a study in wistar rat. *PLoS One* 10:e0138027. 10.1371/journal.pone.0138027 26366995 PMC4569353

[B54] Melgar-Rojas P. Alvarado J. C. Fuentes-Santamaría V. Juiz J. M. (2015b). “Cellular mechanisms of age-related hearing loss,” in *Free Radicals in ENT Pathology*, eds Miller J. M. Prell C. G. Le Rybak L. (Cham: Springer International Publishing), 305–333. 10.1007/978-3-319-13473-4_15

[B55] Mills D. M. Schmiedt R. A. (2004). Metabolic presbycusis: differential changes in auditory brainstem and otoacoustic emission responses with chronic furosemide application in the gerbil. *J. Assoc. Res. Otolaryngol.* 5 1–10. 10.1007/s10162-003-4004-3 14605922 PMC2538367

[B56] Möhrle D. Ni K. Varakina K. Bing D. Lee S. C. Zimmermann U. (2016). Loss of auditory sensitivity from inner hair cell synaptopathy can be centrally compensated in the young but not old brain. *Neurobiol. Aging* 44 173–184. 10.1016/j.neurobiolaging.2016.05.001 27318145

[B57] Niu X. Canlon B. (2002). Protective mechanisms of sound conditioning. *Adv. Otorhinolaryngol.* 59 96–105. 10.1159/000059246 11885667

[B58] Overbeck G. W. Church M. W. (1992). Effects of tone burst frequency and intensity on the auditory brainstem response (ABR) from albino and pigmented rats. *Hear. Res.* 59 129–137. 10.1016/0378-5955(92)90110-9 1618705

[B59] Parham K. Lin F. R. Blakley B. W. (ed.). (2015). “Age-related hearing loss,” in *Geriatric Otolaryngology* (New York: Thieme), 260.

[B60] Pilati N. Ison M. J. Barker M. Mulheran M. Large C. H. Forsythe I. D. (2012). Mechanisms contributing to central excitability changes during hearing loss. *Proc. Natl. Acad. Sci. U.S.A.* 109 8292–8297. 10.1073/pnas.1116981109 22566618 PMC3361412

[B61] Popelar J. Grecova J. Rybalko N. Syka J. (2008). Comparison of noise-induced changes of auditory brainstem and middle latency response amplitudes in rats. *Hear. Res.* 245 82–91. 10.1016/j.heares.2008.09.002 18812219

[B62] Popelar J. Groh D. Pelánová J. Canlon B. Syka J. (2006). Age-related changes in cochlear and brainstem auditory functions in Fischer 344 rats. *Neurobiol. Aging* 27 490–500. 10.1016/j.neurobiolaging.2005.03.001 16464658

[B63] Pukkila M. Zhai S. Virkkala J. Pirvola U. Ylikoski J. (1997). The “toughening” phenomenon in rat’s auditory organ. *Acta Otolaryngol. Suppl.* 529 59–62. 10.3109/00016489709124081 9288269

[B64] Rybalko N. Bureš Z. Burianová J. Popelář J. Poon P. W. F. Syka J. (2012). Age-related changes in the acoustic startle reflex in Fischer 344 and Long Evans rats. *Exp. Gerontol.* 47 966–973. 10.1016/j.exger.2012.09.001 22982446

[B65] Schmiedt R. A. (2010). “The physiology of cochlear presbycusis,” in *The Aging Auditory System*, eds Gordon-Salant S. Frisina R. D. Popper A. N. Fay R. R. (New York, NY: Springer), 9–38. 10.1007/978-1-4419-0993-0_2

[B66] Sergeyenko Y. Lall K. Liberman M. C. Kujawa S. G. (2013). Age-related cochlear synaptopathy: an early-onset contributor to auditory functional decline. *J. Neurosci.* 33 13686–13694. 10.1523/JNEUROSCI.1783-13.2013 23966690 PMC3755715

[B67] Shi X. (2011). Physiopathology of the cochlear microcirculation. *Hear. Res.* 282 10–24. 10.1016/j.heares.2011.08.006 21875658 PMC3608480

[B68] Śliwińska-Kowalska M. Davis A. (2012). Noise-induced hearing loss. *Noise Health* 14 274–280. 10.4103/1463-1741.104893 23257577

[B69] Śliwińska-Kowalska M. Zaborowski K. (2017). WHO environmental noise guidelines for the European region: a systematic review on environmental noise and permanent hearing loss and tinnitus. *Int. J. Environ. Res. Public Health* 14:E1139. 10.3390/ijerph14101139 28953238 PMC5664640

[B70] Sprinzl G. M. Riechelmann H. (2010). Current trends in treating hearing loss in elderly people: a review of the technology and treatment options – a mini-review. *Gerontology* 56 351–358. 10.1159/000275062 20090297

[B71] Subramaniam M. Campo P. Henderson D. (1991). The effect of exposure level on the development of progressive resistance to noise. *Hear. Res.* 52 181–187. 10.1016/0378-5955(91)90197-H 2061206

[B72] Subramaniam M. Henderson D. Campo P. Spongr V. (1992). The effect of “conditioning” on hearing loss from a high frequency traumatic exposure. *Hear. Res.* 58 57–62. 10.1016/0378-5955(92)90008-B 1559906

[B73] Syka J. (2002). Plastic changes in the central auditory system after hearing loss, restoration of function, and during learning. *Physiol. Rev.* 82 601–636. 10.1152/physrev.00002.2002 12087130

[B74] Syka J. (2010). The Fischer 344 rat as a model of presbycusis. *Hear. Res.* 264 70–78. 10.1016/j.heares.2009.11.003 19903514

[B75] Tanaka C. Bielefeld E. C. Chen G.-D. Li M. Henderson D. (2009). Ameliorative effects of an augmented acoustic environment on age-related hearing loss in middle-aged Fischer 344/NHsd rats. *Laryngoscope* 119 1374–1379. 10.1002/lary.20244 19418535

[B76] Tavanai E. Mohammadkhani G. (2017). Role of antioxidants in prevention of age-related hearing loss: a review of literature. *Eur. Arch. Otorhinolaryngol.* 274 1821–1834. 10.1007/s00405-016-4378-6 27858145

[B77] Trowe M.-O. Maier H. Schweizer M. Kispert A. (2008). Deafness in mice lacking the T-box transcription factor Tbx18 in otic fibrocytes. *Development* 135 1725–1734. 10.1242/dev.014043 18353863

[B78] Turner J. G. Willott J. F. (1998). Exposure to an augmented acoustic environment alters auditory function in hearing-impaired DBA/2J mice. *Hear. Res.* 118 101–113. 10.1016/S0378-5955(98)00024-0 9606065

[B79] Walling A. D. Dickson G. M. (2012). Hearing loss in older adults. *Am. Fam. Physician* 85 1150–1156. 22962895

[B80] Willott J. F. Turner J. G. (1999). Prolonged exposure to an augmented acoustic environment ameliorates age-related auditory changes in C57BL/6J and DBA/2J mice. *Hear. Res.* 135 78–88. 10.1016/S0378-5955(99)00094-5 10491957

[B81] Willott J. F. Turner J. G. Sundin V. S. (2000). Effects of exposure to an augmented acoustic environment on auditory function in mice: roles of hearing loss and age during treatment. *Hear. Res.* 142 79–88. 10.1016/S0378-5955(00)00014-9 10748331

[B82] World Health Organization (2006). *Primary Ear and Hearing Care: Training Resource.* Geneva: World Health Organization.

[B83] World Health Organization (1997). *Prevention of Noise-Induced Hearing Loss.* Available at: http://apps.who.int/iris/bitstream/handle/10665/65390/WHO_PDH_98.5.pdf?sequence=1&isAllowed=y

[B84] World Health Organization (2017a). *10 Facts on Ageing and Health.* Geneva: World Health Organization.

[B85] World Health Organization (2017b). *Deafness and Hearing Loss.* Geneva: World Health Organization.

[B86] World Health Organization (2018). *Addressing the Rising Prevalence of Hearing Loss.* Geneva: World Health Organization.

[B87] Yamasoba T. Dolan D. F. Miller J. M. (1999). Acquired resistance to acoustic trauma by sound conditioning is primarily mediated by changes restricted to the cochlea, not by systemic responses. *Hear. Res.* 127 31–40. 10.1016/S0378-5955(98)00178-6 9925014

[B88] Yamasoba T. Lin F. R. Someya S. Kashio A. Sakamoto T. Kondo K. (2013). Current concepts in age-related hearing loss: epidemiology and mechanistic pathways. *Hear. Res.* 303 30–38. 10.1016/j.heares.2013.01.021 23422312 PMC3723756

